# Development of three-dimensional prints of arthritic joints for supporting patients’ awareness to structural damage

**DOI:** 10.1186/s13075-017-1234-z

**Published:** 2017-02-10

**Authors:** Arnd Kleyer, Laura Beyer, Christoph Simon, Fabian Stemmler, Matthias Englbrecht, Christian Beyer, Jürgen Rech, Bernhard Manger, Gerhard Krönke, Georg Schett, Axel J. Hueber

**Affiliations:** 1 Department of Internal Medicine 3 – Rheumatology and Immunology, Friedrich-Alexander-University Erlangen-Nürnberg (FAU) and Universitätsklinikum Erlangen, Ulmenweg 18, 91054 Erlangen, Germany; 2 Department of Internal Medicine 3 and Institute for Clinical Immunology, University of Erlangen-Nuremberg, Ulmenweg 18, 90154 Erlangen, Germany

**Keywords:** Arthritis, 3D Printing, Adherence, Bone

## Abstract

**Background:**

Rheumatoid arthritis (RA) and psoriatic arthritis (PsA) result in severe joint destruction and functional disability if left untreated. We aim to develop tools that help patients with RA and PsA to understand and experience the impact of inflammatory joint disease on the integrity of their (juxta-articular) bone and increase adherence to medical treatment. In this study, we used high-resolution peripheral quantitative computed tomography (HR-pQCT) to develop 3D prototypes of patients’ finger joints.

**Methods:**

HR-pQCT (XtremeCT, Scanco) measurements were performed in healthy individuals and patients with inflammatory joint disease, followed by a 3D print using the objet30 printer. Healthy participants (n = 10), and patients (n = 15 with RA and 15 with PsA) underwent a detailed, standardized interview with demonstration of printed joints.

**Results:**

Utilizing HR-pQCT images of metacarpophalangeal (MCP) heads, high quality and exact 3D prints as prototypes were created. Erosions in different sizes and the trabecular network printed in detail were visualized, demonstrating structural reduction in arthritic vs. healthy bone. After demonstration of 3D prints (healthy vs. erosive joint, visual and haptic) 26/39 (66%) participants (including healthy volunteers) were deeply affected, often quoting “shock”. Of the patients with RA and PsA, 13/15 (86%) and 11/15 (73%), respectively, stated that they would rethink their attitude to medication adherence. More importantly, 21/24 patients with RA or PsA (87.5%) expressed that they would have wished to see such 3D prints during their first disease-specific conversations.

**Conclusion:**

Using arthro-haptic 3D printed prototypes of joints may help to better understand the impact of inflammatory arthritides on bone integrity and long-term damage.

**Electronic supplementary material:**

The online version of this article (doi:10.1186/s13075-017-1234-z) contains supplementary material, which is available to authorized users.

## Background

Rheumatoid arthritis (RA) and psoriatic arthritis (PsA) are inflammatory diseases, which lead to joint destruction and functional disability if not adequately treated [1–3]. In RA, autoantibodies such as rheumatoid factor or anti-citrullinated peptide antibodies (ACPA) are typical serologic findings and are strongly associated with erosive disease [4]. Aside from bone erosion, pathognomonic features of RA comprise articular and periarticular bone loss and loss of trabeculation [5, 6]. In PsA no immune factor has been described yet, and clinically joint-associated bone changes include erosive disease and juxta-articular bone remodeling [2, 7–10].

A panel of highly effective and approved immunosuppressive drugs is available to treat inflammation and to avoid joint damage [3]. Despite these treatment opportunities, recent studies demonstrated poor adherence to these drugs [11, 12]. The reasons for this are multifactorial; however, it is mainly influenced by medical beliefs and lack of understanding of the disease [13]. Current tools for patients comprise leaflets and brochures, digital tools such as apps and website content and patient communities. Most of these tools have either not been evaluated scientifically or have not improved adherence when tested in clinical trials [14].

Focusing on the damage induced by inflammatory arthritis, radiographic techniques can be used to visualize bone changes. To date, conventional radiographs of the hands and feet are the gold standard in detecting and monitoring erosions; however, this method captures images only two dimensions and only larger sized erosions are depicted. High-resolution peripheral quantitative computed tomography (HR-pQCT) is approved to determine bone mineral density in human radial and tibial bone [15]. In recent years HR-pQCT has been shown to be a feasible instrument to evaluate bone microstructure such as erosions, osteophytes, articular trabecular network and the cortical border of arthritic finger joints. In contrast to conventional radiographs, HR-pQCT allows three-dimensional visualization of the scanned bone and exact separation of trabecular and cortical bone compartments, at a resolution of 82 μm [6, 16–19]. Generation of computed tomography (CT) images is complex, and not easily usable for day-to-day patient contacts, because 3D features are not available on every desktop computer.

Three-dimensional (3D) printing allows prototyping with almost any material. It is a rapid emerging technology, easy to use and quickly accessible. Different printing methods are available, with main principles in common: the generation of a 3D printed prototype model on the basis of a computer-aided design (CAD) model is transformed into a standard triangulation language (STL) file and after transfer to the 3D printer is printed by either polyjet, stereolithography or other procedures [20]. Different materials such as plastic or metal can be used. So far, medical approaches to 3D printing include planning for surgery, implant devices and bioprinting of tissue structures [21–24].

In this experimental study, we tested (1) the feasibility to generate exact 3D printed prototypes of arthritic and healthy joints from cross-sectional HR-pQCT data and in longitudinal HR-pQCT of a patient with RA, captured in a video to explain the development of erosion, and (2) whether patients accept such prototypes and if these lead to improvement in understanding disease.

## Methods

### Patient characteristics and image selection

HR-pQCT (XtremeCT, Scanco) measurements were performed as previously described in other studies [5, 6]. For 3D printing we selected images of healthy individuals and patients with RA at different stages of disease ranging from erosive, destructive disease to non-erosive bone alterations. To explain the course of different types of bone erosions we chose a female, ACPA-positive patient with RA, treated with a biologic disease-modifying antirheumatic drug (DMARD). To test the acceptance and understanding of printed joints in interviews, 40 people were selected comprising 10 healthy participants, 15 patients with RA and 15 patients with PsA. All datasets were pseudonymized.

### Three-dimensional printing of joints from HR-pQCT data

For 3D printing we chose the objet30 Printer from Stratasys due to its extraordinary resolution at 24 μm. As the data source for our concept models we chose HR-pQCT (Xtreme CT, Scanco) images of the metacarpal heads. In contrast to magnetic resonance imaging (MRI), HR-pQCT acquires images at a much higher resolution and depicts bone microstructure [19]. Furthermore, compared to clinical CT the radiation dose is considerably lower. After motion grade assessment using sixfold zoom of the head of the metacarpophalangeal (MCP) joint the respective bone area was contoured and segmented. The segmented bone was used as the source for STL file creation with SCANCO software. Files were exported and repaired with Netfabb basic (Ver. 5.2.1) and Meshmixer (Ver. 10.8.126) and a final 3D printable file was generated and sent to the objet30 platform for 3D printing.

### Movie production

For visualization of erosions and cystic structures that develop during the course of disease one ACPA-positive female patient (50 years of age) with RA was selected with a dataset covering 2 years of disease duration. The cystic erosive structure was artificially cut in a sagittal section using Netfabb basic (Ver. 5.2.1) and Meshmixer (Ver. 10.8.126) software. The video file was captured using the Canon EOS 5D Mark 3 and Canon EOS 650D and was modified using Adobe Premiere CC 2014.

### Interview study design

To study the acceptance of 3D prints, 10 healthy participants (HP) (6 women and 4 men, average age 43.6 years), 15 patients with RA (11 women and 4 men, average age 56 years, average disease duration 14.3 years) and 15 patients with PsA (9 women and 6 men, average age 50 years, average disease duration 11.9 years, all of a Caucasian ethnic background) were interviewed. Healthy participants were informed about the diseases, using a national society brochure about arthritis.

**Fig. 1:**
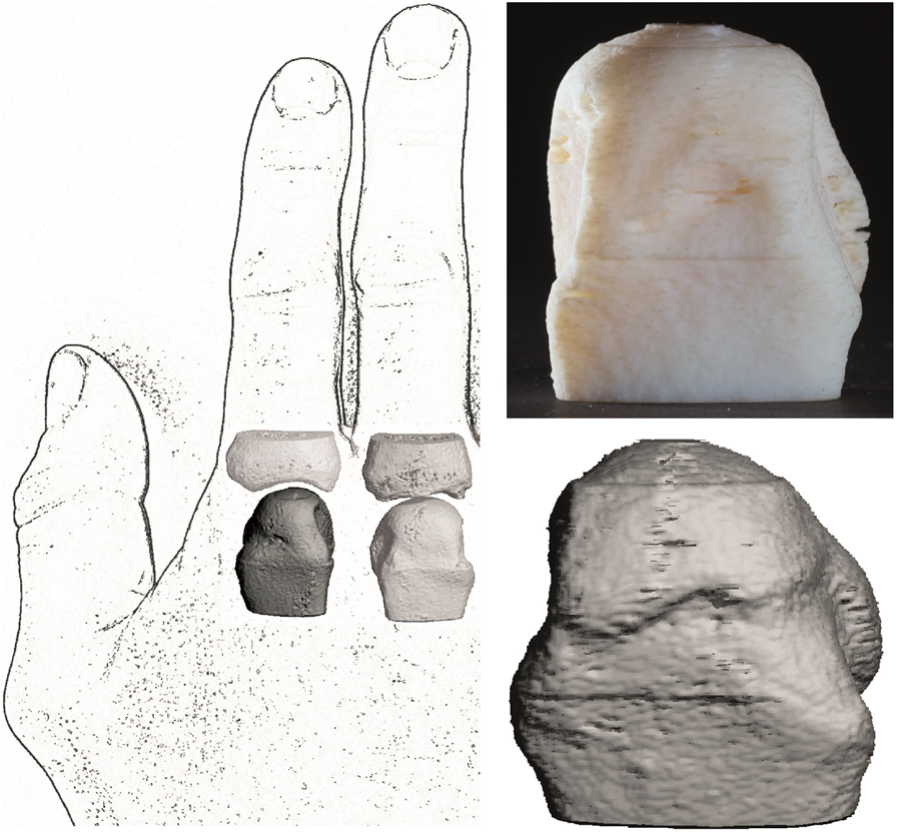
Three-dimensional (3D) model prototype of the second metacarpophalangeal (MCP2) joint. Anatomical structure of MCP joints is depicted (*left panel*). *Lower right panel* demonstrates high-resolution peripheral quantitative computed tomography 3D reconstruction of a healthy MCP2 joint. *Upper right panel* 3D high-resolution model printed using a polyjet procedure (objet30, Stratasys)

**Fig. 2:**
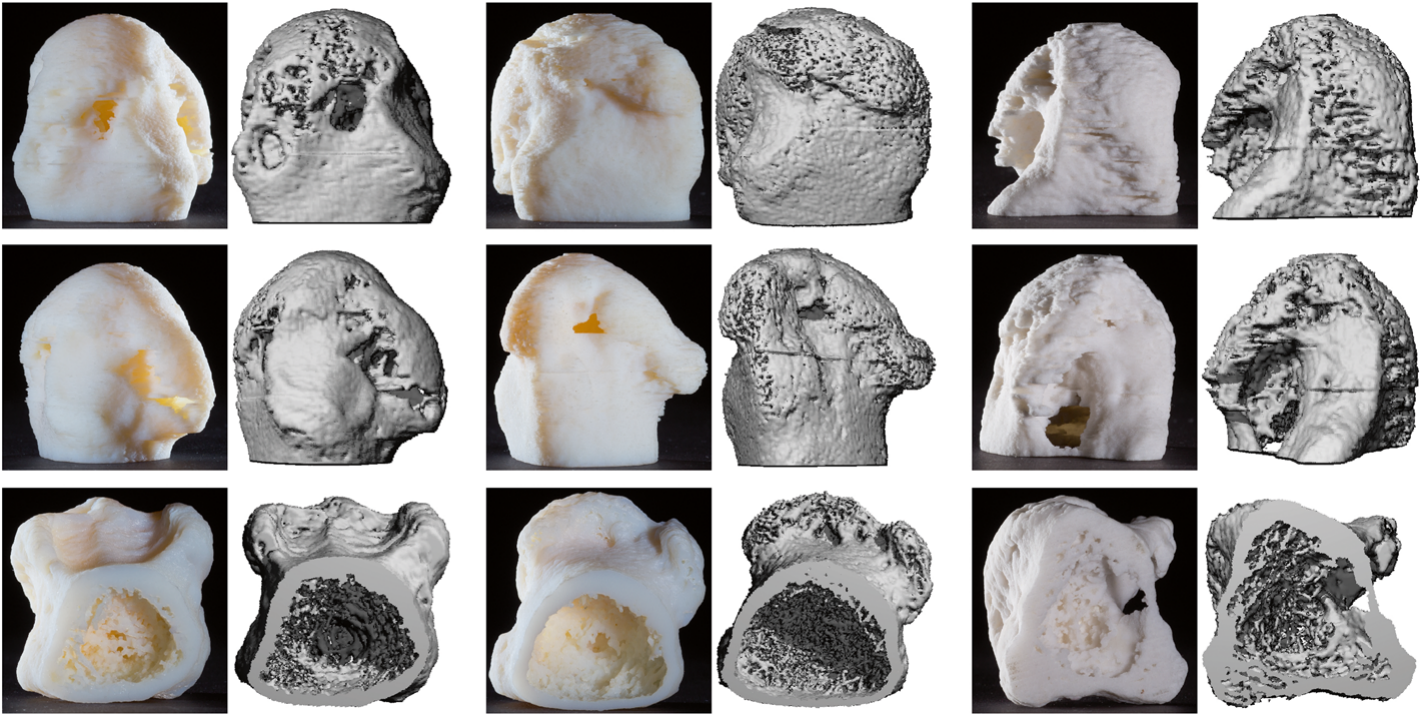
Three-dimensional (3D) visualization of rheumatoid arthritis pathological change. Three individual metacarpophalangeal (MCP) heads in patients with rheumatoid arthritis are presented demonstrating erosive disease and osteophytes (*left*, *middle*, *right panel*). Further, loss of trabecular structure (*middle panel*, *bottom row*) and increased porosity are visible in all examples (in comparison to Fig. 1). 3D models exactly depict 3D high-resolution peripheral quantitative computed tomography reconstructions

The interviews were held in German, were audio-recorded, transcribed verbatim, translated into English and analyzed (for the complete interview guideline see Additional file 1). Interviews lasted about 10 to 20 minutes and were guided by an interview schedule developed in collaboration with a psychologist. Questions explored participants’ opinions of therapy methods and their reactions to the demonstration of a 3D printed prototype of an erosive MCP joint (Figs. 1 and 2). The joints were also demonstrated as pictures, to allow a comparison. A detailed explanation of the joints was given to every participant afterwards. The coded interview data were analyzed using NVivo10 software.

## Results

### From CT images to production of 3D printed prototypes of finger joints

HR-pQCT images of a healthy intact second MCP (MCP2) head and arthritic joints in ACPA-positive patients with RA (n = 25) were used for prototype establishment. Following file repair, a high-quality and exact 3D model was created (Fig. 1, healthy joint).

For better visualization we chose an enlargement of 3:1 compared to the source data. Figure 2 displays representative printouts of MCP2 heads in three different angles (dorsal/radial/trabecular) in three different patients with pathognomonic bone alterations, for arthritic joints. Different-sized erosions and the trabecular network were visualized in detail, demonstrating a massive structural reduction in arthritic vs. healthy bone. A distinct pattern of localization was detected in erosions. The erosions were mainly found at a region close beneath the radial/ulnar or dorsal cartilage area (Fig. 2). No erosions were identified at the palmar location.

### Motional visualization of erosive disease

**Fig. 3:**
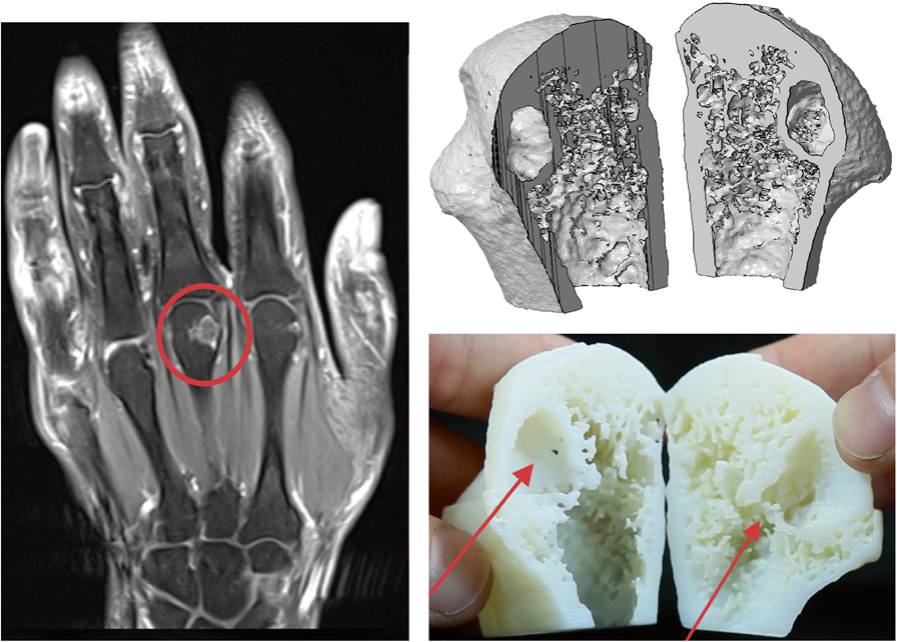
Preparation of a truncated 3D metacarpophalangeal (MCP) head for video presentation. Magnetic resonance imaging (MRI) shows the cystic structure in the third MCP head in a patient with rheumatoid arthritis (*left panel*, *red circle*). In a 3D reconstruction this structure was artificially cut to demonstrate the internal bone formation (*right upper panel*). 3D prints of both halves resemble the cystic structure seen on MRI and computed tomography. Bottom *right panel*: *Left red arrow* shows the MRI corresponded cystic structure; right arrow connection tothe trabecular network. Video motion (Additional file 2) illustrates arthro-haptic experience for patient demonstration



Examining changes in the progression of disease is pivotal for understanding the pathogenesis of RA. Using longitudinal follow-up scans of the MCP joints, we observed the development of erosion in a representative patient. Artificial truncation through a cystic structure that defined erosion that was yet to develop separated the MCP head into two parts. This was 3D-printed for demonstration (Fig. 3) and filmed for motional visualization (Additional file 2). Interestingly, the print depicted two types of erosion. A dorsal porous structured cortical defect with irregularly shaped borders depicted erosion that usually could not be detected on conventional radiographs. In addition, a cystic structure that was also detected by MRI showed contrast enhancement (Fig. 3). At 2-year follow up the cystic structure contained new erosion; however, the borders showed signs of thickening. Thus, this video presented an illustration that was close to reality, of an arthro-haptic experience with a focus on joint architecture and bone changes over time.

**Additional file 2:** Video capture of an artificially truncated MCP head. This video illustrates the making and development of 3D prototypes. Focusing on an artificial cut of the metacarpal phalangeal head, a cystic structure inside the bone, and the bone surface, are demonstrated at two time points. At first the cyst is encapsulated, with no connection to the joint space and dorsal irregular erosion is visualized; over time (2 years) new erosion develops from the cystic structure, with a distinguished pattern compared to the dorsal one. The video also demonstrates the visual experience of 3D prototypes (MPG 26136 kb)


### Perception of 3D printed prototypes

Following the establishment of 3D prototypes we next sought to understand how implementation of 3D joints can be utilized for patient communication. Qualitative interviews were performed in healthy participants who had been informed about inflammatory joint disease. In this setting, this small sample simulates newly diagnosed patients.

After 3D demonstration (healthy joint vs. erosive joint, visual and haptic), healthy participants were asked for their emotional opinion. Nine out of ten healthy participants were deeply affected, often quoting “shock”. Although they had been well-informed earlier, participants did not expect such an impressive impact of the demonstration (Table 1, quotes 1-5). Nevertheless, the demonstration was appreciated by 8 out of 10 participants (Table 1, quotes 6-8). One participant said that it might be too much of a shock and that a series of 3D printed prototypes with more intermediate versions could be superior (Table 1, quote 9).

**Table 1:** Selection of representative interview quotes

Healthy group			
Group and theme	Quote number	Participant	Quote
Theme 1: How does it make you feel to see this?			
	1	005	“Seeing the degeneration is frightening.”
	2	006	“It shocks. I’d do anything to prevent it from worsening.”
	3	005	“It would not scare me off, it would rather wake me up.”
	4	009	“It’s terrible. It clearly shows that the process is irreversible.”
	5	005	“Wow, that’s shocking- in some areas there is nothing left of the bone.”
Theme 2: What do you think about the demonstration of the healthy and the erosive joint?			
	6	005	“To see it from the inside […] to realize what it looks like under my skin- it’s on the one hand macabre, but […] it would affect me more and therefore I would not forget my medication. Because I have now seen what could happen to my joints if I don’t do anything about it.”
	7	006	“You know, sometimes it’s difficult for us as ordinary people to understand what the doctor is trying to tell you. But with this model-it’s the same thing with a car crash: when I see where the damage is, I can come up with something to fix it. I know what to do, how to repair it. Having seen this damage (because of RA and PsA) and the doctor telling me what to do about it- it’s just obvious and not anonymous anymore.”
	8	013	“The diagnosis scares anyway, so I guess it’s better to know what you’re dealing with and what might happen. Being left in the dark is even scarier. And since you can’t change anything about it anymore, you have to cope with it, if you want to stop it from proceeding. So yes, I would prefer being informed with such a 3D model.”
	9	012	“[…] 3 models is a reasonable option, because it shows, that one hasn’t reached the final stage yet, and that one can still do something about it.”
Theme 3: What would be the difference between a computer animation or a 3D model?			
	10	006	“It’s (the 3D model) more real, not as abstract.”
	11	012	“I think that some people don’t take their medication, because they don’t understand it, they don’t have a relation to it and I am sure, that this 3D model could somehow reach patients easier than the more distanced computer animation.”
	12	011	“I believe that especially older patients, who don’t deal with computers that often, might prefer the 3D model.”
Patient group			
Group and theme	Quotation number	Patient number	Quote
Theme 1: What did the diagnosis mean for you?			
	1	2.0	“It was a shock. Embarrassing, that everybody could see the dandruff. I didn’t wear black any longer.”
	2	1.1.4	“I try to put my better hand above the distorted fingers on pictures so that not everybody will see it immediately.”
	3	2.5	“You’re going to laugh, but I was relieved that I finally knew what was going on and a spade was a spade. I was moving like an old man and was simply glad to have found somebody who told me what was happening to me.”
Theme 2: “What do you feel like, being confronted with the 3D print model?”			
	4	2.1	“There is always this fear. If I take a look at the degenerated one, I just think-great, if I don’t watch out, mine will look exactly as degenerated.”
	5	2.2	“I’ve got to be serious. I didn’t think it was that bad.”
	6	2.6	“I’m just glad that my joints haven’t degenerated as far.”
	7	3.3	“I think it might be especially helpful for patients who have just been diagnosed or who would not deal with it reasonably.”
	8	2.0	“The 3D model is simply very close. I appreciate that. Seeing this affects, because I know that it could be me and that it might get worse one day.”
Theme 3: “Would you prefer a series of 3 models, instead of 2, so that the process could be demonstrated in a more precise way?”			
	9	2.5	“I think this would be great, especially for rather sensitive people, who can be sloppy as well and who could benefit from the demonstration.”
	10	1.1.1	“That would be great, because it could wake up, but wouldn’t shock too much all the same.”
Theme 4: “What would be the difference between a computer animation and the 3D print model?			
	11	2.4	“It is always better to hold something than to see it on pictures only. Especially for older people who don’t deal with computers that often.”
	12	2.5	“A 3D model is closer […] and the impression lasts longer. We live in a society that deals a lot with computers and I think we have learnt how to turn out things we don’t want to look at. The print is more impressive, more gripping.”
	13	2.9	“Reality is always better.”
	14	1.4	“It’s just a different feeling, I can hold it and feel it.”
Theme 5: “Would you prefer being confronted with an example joint or your own joint as a 3D print model?”			
	15	2.3	“Seeing my own would motivate me even more. Because I have just seen my very own joint and that is worse than seeing someone else’s.”
Theme 6: “Could this demonstration increase patients’ adherence?”			
	16	1.1.6	“I definitely think that [it would be adherence supportive], yes. If I wouldn’t already take my medicine, I would by now.”
	17	1.1.2	“Well, I think if you’d show this to someone, who doesn’t know much about medicine, you could […] make him understand and take the medication to avoid the degeneration.”
	18	2.6	“Especially if the patient thinks, that the pills are only against the pain and if he doesn’t realize what else is going to happen […], that there is a real, degenerative change in his body and the process is irreversible.”

Questioned about the difference between a 3D model and a computer animation, 9 out of 10 participants stated that they preferred the 3D model. Also the haptic demonstration was more concrete and seemed to emotionally affect participants on a larger scale. The less fictional 3D model could be advantageous, especially for older patients (Table 1, quotes 10-12). When questioned about their preference for either individual 3D joints or a representative model, 6 out of 10 participants expressed a preference for use of their own joints, while 2 participants were indifferent and 2 participants said that an example would be sufficient.

M-any patients with arthritis (19/30 (63%)) reported that they were shocked at the time of the first diagnosis and that the disease severely influenced their lives (Table 1, quotes 1-3). Being confronted with the 3D printed prototype, more than half of the patients were also deeply affected (17/29 (58.6%)), whereas another 7/29 (24%) valued the illustration demonstrated. Although most of them had seen schematic pictures of joint disease before, the direct comparison between the healthy and the erosive model, and the haptic experience, had an emotional impact on most of the patients (Table 1, quotes 4-8). Following the feedback from healthy participants on the advantages of a 3D model series (addition of an intermediate erosive disease model to the healthy and very erosive disease model), 11/11 patients with RA and 9/12 (75%) patients with PsA believed that a series of three models would have better effects (Table 1, quotes 9-10).

Comparing the difference between a 3D model and a computer animation, participants clearly preferred the 3D model (12/15 (80%) of the patients with RA and 10/15 (66%) of the patients with PsA) (Table 1, quotes 11-14). The possibility of touching the models, feeling the erosions and being able to turn it, was very important for internalization according to 22/30 (73%) patients (quotes 13, 14): 8/15 (53.3%) of the patients with RA and 8/12 (66.6%) of the patients with PsA indicated they would appreciate seeing their own joint as a 3D model. Some emphasized that it might increase adherence, as patients are personally confronted (Table 1, quote 15). Of the patients with RA, 13/15 (86%) and 11/15 (73%) of the patients with PsA stated that they would rethink their attitude to medication adherence after being confronted with the 3D printed prototypes (Table 1, quotes 16-18). More importantly, 21/24 (87.5%) of patients with RA or PsA said that they would have wished to see such 3D prints during their first disease-specific conversations. Thus, these interviews endorse the use of 3D prints for patient information and could have an impact on medication adherence.

## Discussion

In this study, we introduced 3D printing in the context of rheumatology and inflammatory arthritis. First, we demonstrated the feasibility of bone prototyping using the combination of HR-pQCT and a desktop 3D printer. We created individual joint models with an extremely high resolution, which depict the trabecular network and erosive changes. Second, we demonstrated erosive change and development of erosions over time. Last, we tested patients’ reactions and their views on future use of 3D prints in patient-doctor communication.

In the current study a poly-jet modeling procedure was used. Also stereolithographic 3D printers achieve high-resolution 3D prints in the range of microns (a set of 10 individual joints were printed as proof of concept, data not shown). The printing material allowed the creation of very durable samples. Printing took 4 to 8 hours depending on the size of the prototype, the resolution and the type of printer. Although 3D STL file creation of joints was simple due its implementation in the HR-pQCT software, moderate effort was necessary to repair and correct the file for error-free printing. Modern platforms already provide STL files for various objects (e.g. thingiverse.com). Also bones such as the tibia or full hands are available on such systems. However, lack of resolution and pathologic changes negate the utility of the files offered for demonstration.

Theoretically and in practice, other imaging 3D files could also be printed, such as in MRI or 3D ultrasound. Disadvantages are limited resolution (MRI) and lack of intra-osseous structure (ultrasound). Although the technical setup already exists, the realization demands a certain program user skill [25]. We predict that in due course future technical compound and software connections will be able to print different medical 3D imaging files by plug-and-play setups [20]. As a prototype example, we were able to visualize different types of erosions, which cannot be seen on conventional radiographs due to the anatomical distribution and small size. Demonstrating the formation of erosion from an MRI contrast-enhanced cystic structure depicts the power of this approach to exactly localize and quantify newly occurring structural damage in patients with arthritis.

In the patient interviews, the limitation of this study was the lack of comparison of 3D prints with 3D images or computer animations. Although we asked about participants preferences, we did not show the alternatives. Furthermore, we did not include a comparator in this study.

Focusing on feasibility, individualized 3D prints demand the image of the individual patient. HR-pQCT imaging is nearly as cost-intensive as common CT imaging. With the further file correction and print processing this process exceeds the simple presentation of conventional radiography and/or ultrasound.

Another drawback for individualized prints is the limited access to HR-pQCT. However, using showcase 3D prints can provide a cheap solution (approximately 1–10 €) demonstrating different stages of diseases on representative joint constructs. In this study a qualitative approach was chosen to gather crucial points from the patient’s perspective. This limitation of lack of quantifiable data needs to be addressed in further studies. Despite the abstract visualization of enlarged MCP joints, patients recognized the prototypes as bone models and appreciated the added value for future patient-doctor communication. We experienced frequent requests for our 3D models to be used in daily practice after demonstration to rheumatologists.

## Conclusions

This manuscript provides a new approach utilizing 3D printing techniques to demonstrate joint disease. Moreover, we were able to prototype reconstructions of early to late-stage destruction of the finger joints. In summary, using individual arthro-haptic joint constructs will address a gap in patient education and disease understanding. In the future, we will set up a platform for downloadable 3D joints for home/office-based printing.

## Additional files

Additional file 1: Interview guideline. (DOCX 18 kb)

## Abbreviations

3D: 3-Dimensional
ACPA: Anti-citrullinated peptide antibodies
CAD: Computer aided design
CT: Computed tomography
HR-pQCT: High-resolution peripheral quantitative computed tomography
MCP: Metacarpophalangeal
MRI: Magnetic resonance imaging
PsA: Psoriatic arthritis
RA: Rheumatoid arthritis
STL: Standard triangulation language
