# Supplementary material for: Development of three-dimensional prints of arthritic joints for supporting patients’ awareness to structural damage
Source: Arthritis Res Ther. 2017 Feb 10;19:34. doi: 10.1186/s13075-017-1234-z (PMC5303254; doi:10.1186/s13075-017-1234-z)
Supplement: Additional file 1: — Interview guideline. (DOCX 18 kb) [file 13075_2017_1234_MOESM1_ESM.docx]

# Interview guideline - High resolution 3D prototypes of arthritic joints (Kleyer et. al.)

Healthy group

Questions:

1. What do you know about RA/PsA?

(presenting a DGRh brochure)

1. Would you be adherent and regularly take your medicine?
2. Would it be hard for you to regularly take your medicine? If so, is there anything that could alleviate the taking of medicine?
3. Which consequences could you imagine, if you did not take the medicine?
4. How would the diagnosis affect your everyday life concerning private and professional life?
5. Do you think medical care is essential? If so, what would you want your doctor to do/ to be like?

***(presentation of two 3D models)***

1. What do you think this represents?

***(explanation afterwards)***

1. How does it make you feel to see this? (***Theme 1***)
2. Imagine you were diagnosed RA/ PsA. Could this model help you with anything?
3. Imagine this would be your joint. How would you feel about it? (***Theme 1***)
4. What do you think about the demonstration of the healthy and the erosive joint? (***Theme 2***)
5. Can you imagine what the joint would look like if the medicine was taken precisely?
6. What would be the difference between a computer animation or a 3D model? (***Theme 3***)
7. Would it make a difference if you were shown your own joint as a 3D model or an example?

Patient group

Questions:

1. Do you suffer from RA/PsA? What did the diagnosis mean to you? (***Theme 1***)
2. Do you take your medicine regularly?

2.a Is there any other part of therapy besides the medication? If yes, please list…

1. How does the medicine influence your everyday life?
2. In your opinion, are there possibilities to support patients in taking their medication regularly?

***(presentation of two 3D models)***

1. What do you think this represents?

***(explanation afterwards)***

1. Why do I show you this printed model of a joint?
2. What do you feel like, being confronted with the 3D print model? (***Theme 2***)
3. What do you feel if you compare the healthy model to the destructed one?
4. How does this affect you?
5. How does it offer you a better understanding of the disease?
6. Would you prefer a series of 3 models, instead of 2, so that the process could be demonstrated in a more precise way? (***Theme 3***)
7. Let’s pretend you wouldn’t be adherent. Would you think about your attitude towards medication after having seen the 3D print models?
8. What would the joint look like when medication is taken properly?
9. Would you have appreciated this kind of information after diagnosis?
10. What would be the difference between a computer animation and a 3D print model? (***Theme 4***)
11. Would you prefer being confronted with an example joint or your own joint as a 3D print model? (***Theme 5***)
12. Which kind of additional information would you appreciate- is there anything that could help you to better understand the diagnosis?
13. Could this demonstration increase patients’ adherence? (***Theme 6***)
